# Pathogenic *Bacillus anthracis *in the progressive gene losses and gains in adaptive evolution

**DOI:** 10.1186/1471-2105-10-S1-S3

**Published:** 2009-01-30

**Authors:** GX Yu

**Affiliations:** 1 Dept. of Biological Science and Dept. of Computer Science, Boise State University, Boise, Idaho 83725, USA

## Abstract

**Background:**

Sequence mutations represent a driving force of adaptive evolution in bacterial pathogens. It is especially evident in reductive genome evolution where bacteria underwent lifestyles shifting from a free-living to a strictly intracellular or host-depending life. It resulted in loss-of-function mutations and/or the acquisition of virulence gene clusters. *Bacillus anthracis *shares a common soil bacterial ancestor with its closely related bacillus species but is the only obligate, causative agent of inhalation anthrax within the genus Bacillus. The anthrax-causing *Bacillus anthracis *experienced the similar lifestyle changes. We thus hypothesized that the bacterial pathogen would follow a compatible evolution path.

**Results:**

In this study, a cluster-based evolution scheme was devised to analyze genes that are gained by or lost from *B. anthracis*. The study detected gene losses/gains at two separate evolutionary stages. The stage I is when *B. anthracis *and its sister species within the Bacillus cereus group diverged from other species in genus Bacillus. The stage II is when *B. anthracis *differentiated from its two closest relatives: *B. cereus and B. thuringiensis*. Many genes gained at these stages are homologues of known pathogenic factors such those for internalin, *B. anthracis*-specific toxins and large groups of surface proteins and lipoproteins.

**Conclusion:**

The analysis presented here allowed us to portray a progressive evolutionary process during the lifestyle shift of *B. anthracis*, thus providing new insights into how *B. anthracis *had evolved and bore a promise of finding drug and vaccine targets for this strategically important pathogen.

## Background

### Genome reduction and gene acquisition in adaptive bacterial evolution: Two sides of coins

Sequence mutations represent a driving force of adaptive evolution in bacterial pathogens. They allow the pathogens to survive and prosper within the host immune systems and to develop unique host specificity [1-4]. It is especially evident in reductive genome evolution where bacteria underwent dramatic lifestyles shifting from a free-living to a strictly intracellular or host-associated life [5-7]. It results in loss or modification-of-function mutations [8]. The results are a loss of many of the genes and the reduction of bacterial genome size [8,9]. The acquisition of virulence gene clusters is another effective strategy in the adaptive evolution of bacterial pathogens from non-pathogenic ancestors [10,11].

The anthrax-causing bacillus species experienced the similar lifestyle changes and likely followed compatible evolution paths. *B. anthracis *shares a common soil bacterial ancestor with *B. cereus*, *B. thuringiensis *and other closely related bacillus species but it is the only obligate, causative agent of inhalation anthrax within the genus Bacillus [12,13]. The bacteria exists in the environment as weatherproof, dormant spores [14] that germinate only after being picked up by macrophages. From there, the *B. anthracis *spores are carried through lung tissue to the regional lymph nodes. During this process, the spores survive and germinate into vegetative bacilli [15,16]. The vegetative bacteria then multiply and penetrate into the blood circulation by disrupting the macrophages, leading to massive septicemia [17]. The dramatic lifestyle change from a soil bacterium to an obligate pathogen would put *B. anthracis *and its genome under a strong selective pressure. This study addressed questions on what occurred in gene content during the adaptive evolution and how they impacted on the pathogenesis of *B. anthracis*.

Comparative analyses of completely sequenced genomes of the closely related Bacillus organisms have offered a unique opportunity to answer these questions. With such comparison, Read *et al*, 2003 were able to show that *B. anthracis *had a reduced capacity for sugar utilization and an expanded array of iron-acquisition genes when compared with *B. subtilis *[18]. Otherwise, almost all putative chromosomal virulence and surface proteins of *B. anthracis *were shown to have homologues in *B. cereus*. Rasko *et al*, 2004 showed that *B. cereus *ATCC 10987, a non-lethal dairy isolate in the same genetic subgroup as *Bacillus anthracis*, contained a single large plasmid [19]. Interestingly, it has similar gene content and genome structure to *B. anthracis *pXO1 but lacks the pathogenicity-associated, anthrax lethal and edema toxin complex genes. Recently, Han *et al, 2006 *revealed more systematic differences between *B. anthracis *and its closest relatives: *B. cereus and B. thuringiensis *[20]. This genome has complete gene sets that are necessary to encode tripartite lethal toxin and polyglutamic acid capsule; moreover, its flagellar genes are the most fragmental and functional PlcRs are absent. The analyses thus provided a basic understanding of molecular mechanisms of evolution and pathogenesis.

In this study, a cluster-based evolution scheme was devised to analyze genes that are gained by or lost from *B. anthracis*. The section of methods and materials has a detailed description about the scheme. Briefly, a reference genome (RG) was chosen and compared via Blast analysis to all other Bacillus genomes, named target genomes (TG). Genes that are present at RG but absent at TG were identified. This led to a gene absence/presence matrix with genes as rows and TGs as columns, which is then subjected to clustering analysis to identify lineage-specific gene losses or gains. In this study, *B. anthracis *Ames Ancestor 0581 was chosen as the RG to identify genes that were gained by *B. anthracis*, *B. cereus *ATCC 10987 and *B. licheniformis *ATCC 14580 as RG for those that were lost. *B. cereus *ATCC 10987, another species in the Bacillus cereus group, is one of the closest species to *B. anthracis *while *B. licheniformis *ATCC 14580, belonging to Bacillus subtilis group, is more remotely related. The gene losses defined from the two organism, we hypothesized, would represent two different stages critical for the evolution of *B. anthracis*. This paper presented the analysis. Overall, the analysis illustrated a progressive evolution behind *B. anthracis*: genes were lost and gained selectively, which, we hypothesized, could be one of the main evolutionary forces that have driven *B. anthracis *to become an effective anthrax pathogen.

## Materials and methods

### Genome sequences

Reference sequences of 12 bacillus genomes were downloaded from the National Center for Biotechnology Information (NCBI) in June 2006 [21]. Among these are three for *B. anthracis (B. anthracis *Ames, *B. anthracis *Ames Ancestor 0581, and *B. anthracis *str. Sterne), three for *B. cereus *(*B. cereus *ATCC 10987, *B. cereus *ATCC 14579, and *B. cereus *E33L), two for *B. licheniformis *(*B. licheniformis *ATCC 14580 and *B. licheniformis *DSM_13 ATCC 14580), and one for each species of *B. subtilis *(*B. subtilis *subsp. Subtilis str. 168), *B. thuringiensis *(*B. thuringiensis *konkukian str. 97-27), *B. halodurans *(*B. halodurans *C-125) and *B. clausii *(*B. clausii *KSM-K16).

### Analysis of genes losses/gains

The analysis came with a three-step procedure. First, a reference genome (RG) was selected, which were then compared with a set of target genomes (TGs) through BLAST analysis. Under the default E-value of 1e-4, any RG genes with no TG homologues were defined as ***ab ***(***ab***sence) genes. The distribution of ***ab ***genes of all RG-TGs pairs was then summarized as an **m**-by-**n **matrix where **m **is the number of ***ab ***genes (rows) and **n **is the number of TGs (columns). Values of the matrix are either "0" or "1" where "1" indicates that genes present in RG but absent in TG for given TG-RG pairs and "0" indicates the genes that are present in both TG and RG. Second, clustering analyses were performed on the data matrix using Cluster (version 3.0), an open source clustering software implemented for gene expression data analysis [22]. Following parameters were chosen in the analysis to give best possible results: Hierarchical, cluster on rows (gene) and columns (genomes); Spearman Rank Correlation; and Single linkage. The last step is to visualize trees from the clustering analysis with Java TreeView [23] to identify lineage-specific gene losses/gains. In this analysis, we chose *B. anthracis *Ames Ancestor 0581 as a RG for the lineage-specific genes gained by *B. anthracis*, *B. licheniformis *ATCC 14580 and *B. cereus *ATCC 10987 as the RG for the lineage-specific genes losses.

### Function classifications

For a better illustration, species-specific genes lost/gained were further characterized based on COG assignments inherited from NCBI genome annotations.

### Calculation of selective gene losses/gains

To determine selective gene gains/losses during the adaptive evolution, the following formula was used to evaluate the occurrences of gene lost/gained among COG function groups:

LGpnt_COGi _= lg_COGi_/LG_COG _* 100;

While LGpnt_COGi _is the percentage of COG occurrences in genes lost/gained, lg_COGi _is the number of genes lost/gained from the i^th ^COG and LG_COG _is the total number of genes lost/gained that have COG assignments in given genomes. At the same time, a similar formula was used to calculate the distribution of the COG occurrences in genes over the entire genomes:

pnt_COGj _= x_COGj_/X_COG _* 100;

While pnt_COGj _is the percentage of COG occurrences, x_COGj_is the number of genes in the j^th ^COG and X_COG _is the total number of genes that have COG assignments. The selective gene losses/gains were determined by comparing the LGpnt_COGi _with pnt_COGj_. Genes were considered to be selectively lost or gained if LGpnt_COGi _is greater than pnt_COGj _at least two fold where i = j.

### Verifications of *B. anthracis*-specific sequence variants

Additional nine Bacillus genomes were downloaded to investigate whether the conclusions about the *B. anthracis*-specific gene changes can be extrapolated to Bacillus organisms beyond the initial genome sets. The new genomes include *B. weihenstephanensis *KBAB4, *B. cereus *subsp. cytotoxis NVH 391–98, *B. thuringiensis *str. Al Hakam, which are available only recently at the NCBI genome site [21]. Also included are six other *B. anthracis *genomes from the Comprehensive Microbial Resource (CMR) [24]: *B. anthracis *A0039, *B. anthracis *A0402, *B. anthracis *str. Kruger B, *B. anthracis *Vollum, *B. anthracis *A0071 Western North America, and *B. anthracis *strain A2012. All genomes downloaded from NCBI are completely sequenced and fully annotated but those from CMR vary in status in terms of sequencing and annotations. For example, the sequence of *B. anthracis *A0039 is listed as unfinished in sequencing with 5634 annotated genes/proteins. In contrast, the sequence of *B. anthracis *strain A2012 is listed as complete in sequencing but with only 330 annotated genes/proteins.

## Results and discussion

### Gene gains at stages

**Figure 1:**
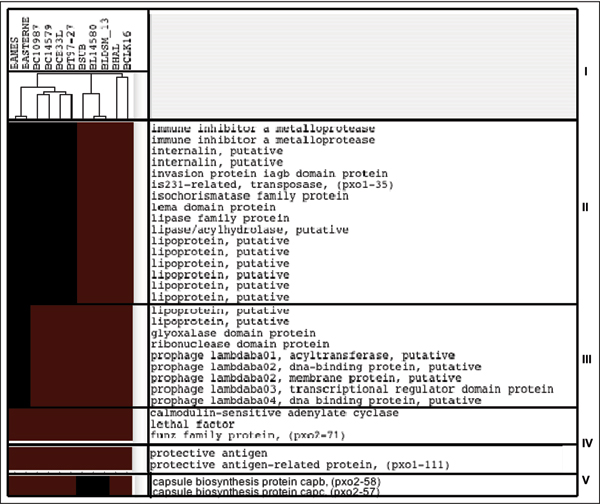
**The clustering analysis of genes gained by *B. anthracis***. The clustering analysis of *B. anthracis*-specific genes that were gained by *B. anthracis*. Genes included are those with obvious function annotations. Colour scheme: red stands for the absence of the genes and black for the presence. Note that genomes in the tree are labelled by their abbreviations: BAMES stands for *B. anthracis *Ames, BANSTR for *B. anthracis *Ames Ancestor 0581, BASTERNE for *B. anthracis *str. Sterne, BC10987 for *B. cereus *ATCC 10987, BC14579 for *B. cereus *ATCC 14579, BCE33L for *B. cereus *E33L, BT97-27 for *B. thuringiensis *konkukian str. 97-27, BL14580 for *B. licheniformis *ATCC 14580, BLDSM_13 for *B. licheniformis *DSM_13 ATCC 14580, BSUB for *B. subtilis *subsp. Subtilis str. 168, BHAL for *B. halodurans *C-125 and BCLK16 for *B. clausii *KSM-K16

The study chose the *B. anthracis *Ames Ancestor 0581 as reference genomes (RGs) and then compared with other 11 bacillus genomes (TG). With an E-value of 1e-4, the comparisons of TG-RG resulted in a gene absence/presence matrix. Clustering analysis on the matrix obtained a phylogenetic (species) tree that reflects evolutionary relationships of these Bacillus species [25]. Genomes of *B. anthracis*, *B. cereus*, and *B. thuringiensis *(the Bacillus cereus group), genomes of *B. subtilis *and *B. licheniformis *(the Bacillus subtilis group), and those of *B. halodurans*, and *B. clausii *were clustered in three separate phylogenetic clades (Fig. 1.I). Under this evolutionary frame, we discovered varieties of gene clusters, which may represent genes gained at two main evolutionary stages. The stage I is when *B. anthracis *and its sister species within the first phylogenetic clade diverged from bacillus species in the other two phylogenetic clades (Fig. 1.II). The stage II is when *B. anthracis *differentiated from *B. cereus and B. thuringiensis*, its closely related, non-anthrax species within the *B. anthracis*, *B. cereus*, and *B. thuringiensis *clade (Fig. 1.III &1.IV). In following sections, we illustrated genes gained at each stage and highlighted those that might have potential impacts on the pathogenesis of *B. anthracis*.

**Figure 2:**
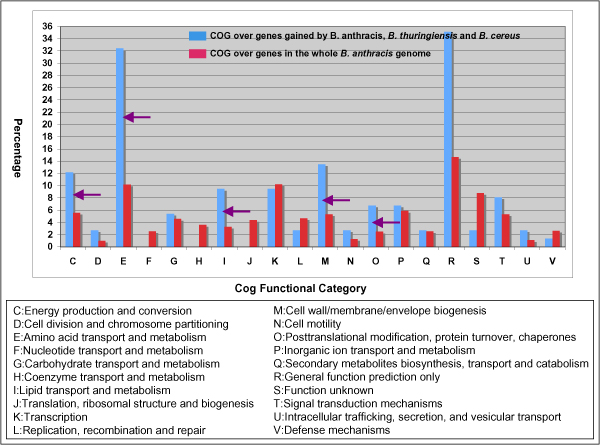
**Comparative display of the COG occurrences of genes gained at stage I over those in the entire genome of *B. anthracis *Ames Ancestor 0581**. Comparative display of the COG occurrences of genes gained at stage I (blue bars) over those in the entire genome of *B. anthracis *Ames Ancestor 0581 (red bars). The Y-coordinate represents the percentage of the gene occurrences in given COGs. Purple arrows represent those with the selective gene gains.

Stage I is corresponding to a cluster of 717 genes that are present only in the genomes of the *B. anthracis*, *B. cereus*, and *B. thuringiensis *clade. Compared with pathogenic species in this clade, bacillus species in other two are non-pathogenic. *B. licheniformis *and *B. subtilis *belong to the *Bacillus subtilis *group and are commonly found soil bacteria. *B. halodurans *and *B. clausii *are widely distributed alkali-tolerant bacillus species. Analysis of the 717-gene cluster indicated that the divergence of the *B. anthracis*, *B. cereus*, and *B. thuringiensis *clade from other non-pathogenic clades appeared to be a critical point in the adaptive evolution of *B. anthracis*. Many genes gained in this stage have homologues of pathogenic importance. Among them, genes in Amino acid transport and metabolism [E] is the most dominant gene group gained at this stage (Fig. 2). It includes 10 genes that encode oligopeptide ABC transporter, oligopeptide-binding proteins, a gene group that are required for bacterial growth at low temperature and involved in intracellular survival [26].

Cell wall/membrane/envelope biogenesis [M] is the second one. It covers, among others, genes for two internalin proteins, invasion protein iagB domain protein, and S-layer protein. The acquisitions of internalin proteins confers the pathogen ability to cross significant host barriers for entering, reside in, and multiply in phagocytic and non-phagocytic cells [27-30]. The Invasion protein iagB domain protein has homologues that are involved in invasion of HeLa cells by *Salmonella enterica *subsp. enterica ser. Typhi [31]. The S-layer protein, together with 13 other S-layer proteins that were classified into the unknown COG group, is one of the largest gene groups gained at this stage. The S-layer proteins are generally known to be self-assembled into a supramolecular structure enclosing the bacterial cells. The structure strategically positions to interact with the tissues and body fluids of the host and contributes to the outcome of a host-parasite interaction. In the fish-pathogenic bacterium *Aeromonas salmonicida*, the array of the surface proteins, for example, dramatically enhances the virulence of the bacterium [32]. In the pathogenic strains of *Aeromonas hydrophila *and *Aeromonas veronii *biotype sobria, the surface-exposed and non-surface-exposed epitopes of the S-layer protein provide antigenic diversity [33].

The cluster furthermore includes homologues of genes known to encode the enhancin family protein [COG unsigned], two non-haemolytic enterotoxins [COG unsigned], an immune inhibitor A metalloprotease protein [S], two microbial collagenase proteins [R], up to 27 lipoprotein-coding genes [COG unassigned]. All the genes are related to pathogenesis of infectious agents if not directly to the bacterial pathogen. The enhancin, found in baculoviruses, is a metalloprotease that can boost viral infectivity by degrading the mucin layer surrounding insect guts [34]. The immune inhibitor A metalloprotease enhances virulence in insects through the cleavage of bacteriocidal lectins [35]. The homologues of non-haemolytic enterotoxins are known to be involved in *B. cereus *and *B. thuringiensis *pathogenesis [18]. The lipoprotein, the largest gene group gained at this stage, is a diverse group of proteins with covalent lipid modifications by fatty acids and other lipid moieties. The lipid-modified proteins often play diverse roles from surface adhesion to the translocation of virulence factors into the host cytoplasm. MxiM, a lipoprotein of the type III secretory pathway in *Shigella flexneri*, for example, is important for translocation of invasions [36], and MAA1 of *Mycoplasma arthritidis*, a surface-exposed lipoprotein, is required for adherence to joint tissues early in the infectious process [37]. From that, we were reasoning that gene gains at this stage would build crucial genetic foundations that allowed *B. anthracis *to evolve later as an anthrax pathogen that can enter, survive and replicate within the hash host cell systems.

Stage II includes two clusters with 83 (Fig. 1.III) and 93 genes (Fig. 1.IV) respectively. Since the clusters represent the difference between *B. anthracis *and its closely related, non-anthrax Bacillus species: *B. cereus and B. thuringiensis*, the acquisition of these genes by *B. anthracis *likely represents a recent event in the bacillus evolution. Between two clusters in this stage, genes in 83-gene cluster are present in all genomes of *B. anthracis*, thus specific to *B. anthracis*. The majority of them have no functional annotations except for those encoding two lipoproteins, one glyoxalase domain protein, one ribonuclease domain protein and several prophage lambdaba04 proteins. The genes in the 93-gene cluster are all plasmid genes (pX01 and pX02) and, among them are anthrax-factors such as lethal factor, protective antigen-related protein, protective antigen and calmodulin-seneitive adenylate cyclase. Although they are present only in *B. anthracis *Ames Ancestor 0581, we still considered them as *B. anthracis*-specific or anthrax-causing Bacillus pathogen-specific. The reason is that the pX01 and pX02 are considered an integrated part of the anthrax-causing Bacillus pathogen and *B. anthracis *Ames Ancestor 0581 is the only one that has the two plasmids and is pathogenic. While the above three clusters follow the evolution relationships in their gene occurrences, the cluster that contains the capsule biosynthesis protein capC and capB does not (Fig. 1.V). These two virulent factors exist only in *B. Subtilis *subsp. Subtilis str. 168, *B. licheniformis *DSM_13 ATCC 14580, *B. licheniformis *ATCC 14580 and *B. anthracis *Ames Ancestor 0581. While the capsule proteins are encoded by plasmid (pX02) in *B. anthracis *Ames Ancestor 0581, their homologues in the other three genomes are chromosomal, suggesting a usual mode of gene transmission: from chromosome to plasmids or vise verse.

### Analysis of gene losses

We chose *B. licheniformis *ATCC 14580 as the reference genome to study the lineage-specific gene losses at stage I when *B. anthracis *and its sister species within the *B. anthracis *clade were diverged from Bacillus species in the other two phylogenetic clades. The analysis found a total of 103 genes that are absent in the genomes within the *B. anthracis*, *B. cereus and B. thuringiensis *clade but present in all the genomes from other two non-pathogenic clades. Among them, carbohydrate transport and metabolism (G) is the most predominant functional group, including genes encoding two pectate lyases, polysaccharide lyase family 1 proteins, five glycoside hydrolase family proteins and two L-arabinose isomerase proteins. Cell motility (N) is the second, including multiple genes for flagellar components such as flagellar protein FliS, flagellar hook-basal body protein and CheD chemotaxis protein.

The study further selected *B. cereus *ATCC 10987 as the reference genome to study the lineage-specific gene losses at stage II when *B. anthracis *was differentiated from its two closest relatives. The genome was selected for its close kinship with *B. anthracis *and its characteristic plasmid [38]. The plasmid shares a basic skeleton with pX01, one of the virulent factor-associated plasmids in the anthrax-causing pathogen. The analysis found 184 genes lost from *B. anthracis*. Among them, 23 are present in all genomes of *B. cereus *and *B. thuringiensis*, but not in those of *B. anthracis*. Those include two two-gene groups of IS3-family transposase-coding genes, one regucalcin family protein-coding gene, and three lipoprotein-coding genes. The rest of the 184 genes vary in their presence in the genomes of *B. cereus *or *B. thuringiensis *but are consistently absent from those of *B. anthracis*. For example, genes that code three DNA recombinases, three spore coat proteins, and one transcriptional regulator, MarR family protein are present in the genomes of *B. cereus *only; genes that code two type I restriction-modification system, M subunits and acid-soluble spore protein P are present in *B. cereus *ATCC 14579 and *B. cereus *ATCC 10987 only. The detection of the selective gene losses suggested possible roles in the Bacillus evolution and further experiments are necessary for their validations.

### Verification of the gene gains and losses

**Figure 3:**
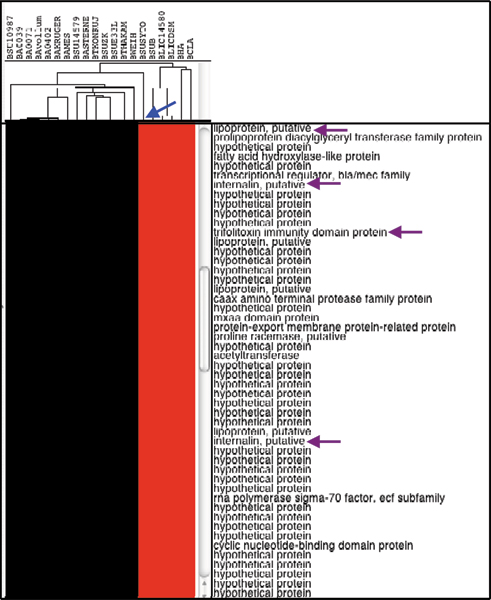
**Display of genes that are absent in the genome of *B. cereus *subsp. cytotoxis NVH 391–98 but present in all other bacillus genomes within the Bacillus cereus group**. Displays of genes that are absent in the genome of *B. cereus *subsp. cytotoxis NVH 391–98 (highlighted by Blue arrow) but present in all other bacillus genomes within the Bacillus cereus group. Purple arrows represent two internalin and other pathogenesis-related genes. The name abbreviations for newly added *B. anthracis *genomes are as followings: BA0039 for *B. anthracis *A0039, BA0402 for *B. anthracis *A0402, BAKROGER for *B. anthracis *str. Kruger B, BAVOLLUM for *B. anthracis *Vollum, and BA0071 for *B. anthracis *A0071 Western North America.

With a set of nine additional bacillus genomes, we examined gene gains and losses at both stages. The results indicated that the inclusion of these genomes did not upset the occurrence patterns of the lineage-specific gene losses and gains except a 119-gene set that were gained at stage I (Fig. 3). These genes are absent in the genome of *B. cereus *subsp. cytotoxis NVH 391–98 but present all other bacillus genomes within the Bacillus cereus group. Among them are those that encode two internalins, cytolysin immunity Cyli domain protein, beta-lactamase II, beta-lactam antibiotic acylase family protein, trifolitoxin immunity domain protein, 11 putative lipoproteins, and nine acetyltransferases. The *B. cereus *strain, isolated in 1998 from an outbreak that caused fatal enteritis, is genetically remote from other B. cereus group strains and highly pathogenic [39,40]. It will be interesting to know how the losses impact on this Bacillus strain and its cytotoxicity.

At the end of this section, we felt that we need to make some clarifications on our newly devised evolution-based scheme and some frequent terms used in the paper. First, we used Blast with a predetermined cutoff to determine gene losses and gains. The results indicated that this is a simplified but valid approach as illustrated above where unique and pathogenically important genes were revealed from this analysis. The drawback is that the results can be compromised if homologous genes exist within the target genomes. One solution is to adjust the threshold. A minor problem is that sequence similarities between orthologs can vary from gene to gene. Alternatively, orthologs can be defined and used in the clustering analysis [41]. Second, we used "gained" to describe genes unique to *B. anthracis *but had no intension to distinguish whether they were actually "acquired" by *B. anthracis *or "pseudogenized" from its compared genomes.

## Conclusion

The analysis presented here allowed us to portray a progressive evolutionary process during the lifestyle shift of *B. anthracis *from a free soil bacterium to an obligate pathogen. Selective gene losses and gains appeared to be one of the main driving forces underlying the adaptive evolution of *B. anthracis*. First, novel genes including those of pathogenic importance were lost/gained when *B. anthracis*, *B. cereus and B. thuringiensis*, the genomes within the Bacillus cereus group, were differentiated from *B. licheniformis *and other *Bacillus *genomes outside the Bacillus cereus group (stage I). Gene losses/gains further occurred in *B. anthracis *when this species diverged from its two closest relatives. Overall, our analysis provided new insights into how *B. anthracis *had evolved and bore a promise of finding drug and vaccine targets for this strategically important pathogen.

## Competing interests

The author declares that they have no competing interests.

## Authors' contributions

GXY is the sole contributor to this paper, covering all research processes from algorithm developments, data collections to data analysis.
